# The impacts of ageing-related changes on prehospital trauma care for older adults: challenges and future directions

**DOI:** 10.3389/fmed.2025.1588927

**Published:** 2025-06-27

**Authors:** Naif Harthi, Steve Goodacre, Fiona C. Sampson

**Affiliations:** ^1^Emergency Medical Services Programme, Department of Nursing, College of Nursing and Health Sciences, Jazan University, Jazan, Saudi Arabia; ^2^Health Research Center, Jazan University, Jazan, Saudi Arabia; ^3^Sheffield Centre for Health and Related Research (SCHARR), University of Sheffield, Sheffield, United Kingdom

**Keywords:** ageing changes, ageing impacts, trauma, prehospital care, older people

## Abstract

The ageing global population presents growing challenges for prehospital trauma care, particularly in addressing the complex needs of older adults. This narrative review explores the impacts of ageing-related anatomical and physiological changes on trauma care in the prehospital setting, with a focus on the challenges they pose for paramedic assessment, triage, and decision-making. These changes affecting the nervous, cardiovascular, respiratory, musculoskeletal, and renal systems that reduce physiological resilience and increase vulnerability to trauma, especially when compounded by frailty, polypharmacy, and comorbidities. The review highlights significant limitations in current trauma triage tools, which often lack sensitivity for identifying serious injuries in older adults and fail to incorporate frailty assessments. Although some protocols, such as the Ohio Trauma Triage Protocol, include geriatric adaptations, traditional tools continue to underperform, contributing to undertriage and suboptimal outcomes. Validated frailty assessment tools, including the Clinical Frailty Scale (CFS), Programme on Research for Integrating Services for the Maintenance of Autonomy (PRISMA-7), and Identification of Seniors at Risk (ISAR), offer promising potential for improving triage accuracy but are not yet routinely used in prehospital practice. Key gaps identified include insufficient paramedic education on ageing-related conditions, limited awareness of age-specific clinical presentations, and a lack of training in applying geriatric assessment tools. To address these issues, the review recommends integrating frailty screening into EMS triage, enhancing geriatric-specific training, and raising paramedic awareness of the physiological and clinical implications of ageing. Future research should investigate paramedics’ behaviours, decision-making processes, and the feasibility of implementing frailty-based triage in the field. These strategies are essential to advancing prehospital trauma care and improving outcomes for the growing population of older trauma patients.

## 1 Introduction

The global population is ageing at an unprecedented rate, driven by advancements in chronic disease management, healthier lifestyles, and improvements in housing and nutrition (1). These factors have significantly increased life expectancy, but they have also contributed to a rise in trauma-related injuries among older adults, creating new challenges for healthcare systems worldwide (2–5). By 2030, 20% of the United States population will be over the age of 65. Similarly, by 2035, 23% of the United Kingdom population will be over 65, with Europe and Australia expecting 30% and 21% respectively by 2050 and 2054 (3, 6–8). Ageing has been identified as a key factor driving increased injury rates, particularly from mechanisms such as falls and road traffic collisions (9, 10). Consequently, older adults now account for 25% of all injury-related hospital admissions in the United States (11). This growing burden on ambulance services and emergency departments is projected to escalate healthcare costs due to higher complication rates and prolonged hospital stays (11–13).

Age-related anatomical and physiological changes further complicate prehospital trauma care for older adults, as they affect nearly all body systems, reducing physiological reserves and increasing susceptibility to injury (14). Paramedics play a critical role as first responders, yet recognising subtle or atypical presentations in older adults, including the influence of polypharmacy and frailty, remains a significant challenge (1, 3). Although geriatric-specific triage protocols have been developed, traditional tools often lack sufficient sensitivity and fail to incorporate frailty assessments, contributing to undertriage and misaligned care decisions (15). This narrative review synthesises existing literature to explore how ageing-related changes influence prehospital trauma care. It examines the physiological mechanisms underlying injury vulnerability in older adults, the limitations of existing triage tools, and the potential utility of integrating frailty assessments into paramedic practice. By outlining the current challenges and opportunities in geriatric trauma management, this review aims to inform future research, improve paramedic education, and ultimately enhance care quality and outcomes for the ageing trauma population.

## 2 Methods

This narrative review integrates existing literature, following the methodological approach detailed in the subsequent subsections:

### 2.1 Review aim and guiding question

This narrative review was conducted to explore the impacts of ageing-related changes on prehospital trauma care in older adults. The review was guided by the question: How do physiological and anatomical changes associated with ageing influence the prehospital management and outcomes of trauma in older adults?

### 2.2 Sources of information

PubMed and Google Scholar were selected for this review due to their extensive coverage of biomedical and health sciences literature and their broad multidisciplinary reach.

### 2.3 Keywords and search strategy

The literature search employed keywords and phrases including “ageing”, “older adults”, “trauma”, and “prehospital”. Relevant articles were identified based on their focus on ageing impacts on prehospital trauma care for older adults. Boolean operators (e.g., AND and OR) were applied to link search terms with their synonyms and to refine the search outcomes.

### 2.4 Time frame and language restrictions

Studies published between 2005 and 2025 were included to capture the most current and relevant evidence. Only publications in English were considered to ensure consistency, clarity, and alignment with the authors’ language proficiency.

### 2.5 Types of literature

The review included relevant peer-reviewed journal articles, systematic and narrative reviews, as well as government reports and guidelines offering published insights into the impacts of ageing changes on prehospital geriatric trauma care.

### 2.6 Screening process

An initial screening of titles and abstracts was carried out to eliminate irrelevant studies. This was followed by full-text reviews and an examination of the reference lists to assess their relevance to the review aim, ultimately resulting in the inclusion of 76 papers. Of these, 36 originated from the United States (3, 9, 10, 16–48), 18 from the United Kingdom (14, 15, 49–64), 5 from Canada (65–69), 5 from Australia (70–74), 4 from Saudi Arabia (1, 75–77), 2 from Germany (4, 78), 2 from Ireland (79, 80), and 1 each from Poland (81), Switzerland (82), Israel (83), and Vietnam (84).

### 2.7 Integration and analysis

The included studies were thematically analysed, with themes developed iteratively to support the overarching aim of improving ambulance clinicians’ understanding of ageing-related impacts and enhancing prehospital trauma assessment and management in this population.

### 2.8 Synthesis of findings

Findings were synthesised narratively in line with the review’s aim, with studies thematically grouped by affected body systems (e.g., nervous, cardiovascular, and respiratory) and stages of prehospital care (e.g., assessment, surveys, and transport). This structure supported a clinically meaningful synthesis and informed evidence-based recommendations to improve paramedic education, awareness, and care for older trauma patients.

### 2.9 Peer review

The data underwent internal review by the co-authors, followed by internal and external evaluations by experts in emergency care. This peer-review process ensured the findings were accurate, clear, and relevant, and were refined to align with current evidence and best practice.

### 2.10 Data and reference management

Mendeley reference management software was employed to systematically record and organise search results, screening procedures, and citations.

## 3 The anatomical and physiological changes associated with ageing

Ageing is characterised by progressive changes across body systems, leading to physiological decline and, eventually, death (14). These changes affect various systems, including the nervous, cardiovascular, respiratory, musculoskeletal, and renal systems.

### 3.1 The nervous system

Ageing impacts both the central and peripheral nervous systems, which can lead to an increased likelihood of injuries or impair communication between paramedics and older patients (57, 75). Age-related changes include vision and hearing impairments, gait disturbances, cognitive decline, and brain alterations (3, 36). These changes heighten the risk of falls and motor vehicle collisions (MVCs), leading to severe injuries (3, 4). For instance, brain shrinkage with age can create bridging vessels between the brain and cranium, increasing the risk of vessel rupture and haemorrhage (81). These changes can also hinder effective communication between caregivers and older patients, potentially preventing the acquisition of critical information regarding medical history, polypharmacy, and specific needs (9, 70).

### 3.2 The cardiovascular system

Ageing impacts the cardiovascular system in multiple ways. Older adults may have reduced cardiac output, an increased reliance on stroke volume, diminished cardiac reserve, inefficient compensatory mechanisms, and cardiovascular diseases, all of which contribute to an increased risk of injuries (9). Reduced vascular elasticity, atherosclerosis, and increased peripheral resistance can result in early development of cardiac shock, conduction defects, and blunted haemodynamic responses to hypovolaemia, masking significant internal bleeding (3). Polypharmacy further complicates cardiovascular care in older adults, as many take medications such as anticoagulants, antiplatelets, angiotensin-converting enzyme (ACE) inhibitors, and beta-blockers. Anticoagulants and antiplatelets can increase the risk of bleeding (43, 44), while ACE inhibitors and beta-blockers may mask hypovolaemic shock or reduce the tachycardic and hypotensive responses to injuries (45, 46).

### 3.3 The respiratory system

Age-related changes to the respiratory system limit its ability to respond effectively to trauma (47). These changes include thoracic wall stiffness, reduced alveolar elasticity, increased airflow resistance, weakened respiratory muscles, and reduced thoracic wall compliance, which all contribute to impaired gas exchange and reduced ventilation-perfusion ratios (48). Older patients are more prone to deteriorate from minor injuries (4). Studies indicate that minimal thoracic injuries can lead to impaired mechanical ventilation, diminished gas exchange, reduced ventilation-perfusion ratios, and early respiratory failure in older individuals (45). Due to age-related changes, older adults also experience more severe and extensive burns compared to younger adults (47). Inhalation injuries are more common in older adults due to reduced respiratory reserve (47). Healthcare providers must therefore enhance their familiarity with age-related changes to accurately assess respiratory injuries in older adults (16).

### 3.4 The musculoskeletal system

Musculoskeletal decline begins as early as age 30 (47). Muscle mass decreases by approximately 4% per decade after age 25 and by 10% after age 50, due to declining anabolic hormones, nutritional deficiencies, malnutrition, and reduced activity levels (47). These musculoskeletal changes include fragile bones, weakened tendons, ligaments, and cartilages (58), which increase the risk of injury and worsen trauma outcomes in older adults (3). With decreased joint stability and increased risk of spontaneous rupture, older adults with osteoporosis are also at a greater risk of fractures from minor trauma due to the loss of substantial trabecular and cortical bone (79). Fractures must be stabilised early, and older patients should be mobilised to prevent complications (47). Research has confirmed that osteoporosis, frailty, sarcopenia, and osteoarthritis in older adults result in a heightened risk of injury and prolonged recovery, leading to disability and organ dysfunction (17).

### 3.5 The renal system

Ageing affects renal function through decreased glomerular filtration rates, diminished tubular function, and reduced kidney mass resulting from a decline in the size and number of glomeruli (18). Clinicians should consider the impact of these renal changes on older patients with trauma: decreased systemic blood flow in an aged renal system can cause a reduction in renal perfusion in the event of low blood pressure, bleeding, shock, or reduced cardiac output (59, 60). This suggests that older patients are less capable of coping with the effects of injury compared to their younger counterparts (61). There is also evidence that urinary issues like urgency, frequency, nocturia, or incontinence are linked to an increased risk of falls (19, 71). Such symptoms can force abrupt changes in daily routines, potentially causing older adults to engage in dangerous activities. Studies have found that fallers are more likely than non-fallers to experience incontinence or frequent urination (62, 84). Furthermore, stress incontinence and nocturnal or daytime urinary frequency are linked to higher fall risks (62). To help prevent falls, healthcare providers can improve physical activity levels, review medications, and mitigate adverse environmental factors (19).

## 4 The impact of low-energy trauma and pre-existing medical conditions in ageing

Older adults are more susceptible to serious injuries not only due to anatomical and physiological changes but also due to the increased risk of sustaining low-energy trauma (3). Frailty in older individuals elevates the likelihood of falls and significant injury, while their physiological reserves and recovery capacity decline with age (63). In the United Kingdom, major trauma is increasingly seen as an issue affecting older adults aged over 50, rather than primarily being a disease of young men (64). Additionally, older patients are more likely to suffer from comorbidities, which further increase mortality and morbidity following trauma (64).

A US study found that patients with fall-related head injuries had a higher prevalence of comorbidities compared to those involved in MVCs (20). Although some conditions such as hypertension, diabetes, cardiac arrhythmias, and fluid or electrolyte disorders were similar in both groups, individuals with fall-related injuries were more likely to have dementia, depression, or Parkinson’s disease (21). Another study found that comorbidities such as cardiac disease or coagulopathies significantly increased mortality in older patients with isolated closed head injuries (22). A third study indicated that head injuries related to falls were more likely to occur in those with pre-existing dementia, whether due to Alzheimer’s or other causes (72). In older patients with traumatic brain injury (TBI), distinguishing between pre-existing dementia and cognitive impairment resulting from the injury can be challenging (21). The presence of pre-existing cognitive impairment complicates the diagnosis of head injuries after trauma (21). These findings underscore that comorbidities and frailty contribute to an increased risk of falls and exacerbate the severity of resulting injuries.

## 5 Impacts of ageing changes on prehospital trauma care for older patients

The ageing process introduces anatomical and physiological changes that affect every stage of prehospital trauma care for older adults, from initial assessment to hospital transport. This section highlights how these changes influence prehospital care and the unique considerations for managing older trauma patients:

### 5.1 Upon arrival at the scene

When arriving at the scene, paramedics follow established guidelines to assess the mechanism of injury and make informed decisions regarding patient transport and care (15). For older adults, the mechanism of injury such as a fall or low-impact trauma—may be more severe than it appears, necessitating a heightened level of vigilance. Data from a study show that injured older victims have higher fatality rates due to low-velocity vehicles than younger victims (23). Additionally, ambulance personnel need to identify prescribed medications, assess for potential elder abuse, and gather information about pre-event status and the circumstances of the injury (3, 24).

Older adults may present unique challenges, such as osteoporosis and kyphosis, which complicate the stabilisation of the cervical spine and positioning for airway management (3). Full-body immobilisation, which is often necessary in trauma care, can also cause respiratory restrictions in older adults, further complicating care (3). These considerations must be factored into paramedic decision-making to prevent secondary injuries and optimise patient outcomes.

### 5.2 During primary survey

In trauma management, paramedics assess Catastrophic haemorrhage, Airway/C-Spine, Breathing, Circulation, Disability, and Exposure (<C>ABCDE) to identify and manage life-threatening conditions (49). In older patients, the physiological changes associated with ageing, including altered compensatory mechanisms, make recognising and managing life-threatening conditions more challenging (3). During haemorrhage control, ageing changes influence cardiovascular responses to bleeding, which, combined with beta blocker use, may make recognising shock more difficult (3, 25, 36, 46). Antithrombotic medications used by older adults further complicate the management of bleeding (9, 50). These challenges are important to be considered proactively by ambulance workers.

Maintaining the airway and facilitating effective breathing can be complicated by ageing-related issues, such as reduced mouth opening due to arthritis and ineffective ventilation owing to edentulism (3, 48). Age-related changes in the respiratory system, including weakened muscles and reduced compliance of the thoracic wall, further diminish respiratory reserve (3, 48). Additionally, older adults have decreased cardiac reserve and peripheral vascular changes, which may mask hypovolaemia and delay its recognition (3, 48). Consequently, traditional indicators, such as blood pressure and heart rate, may be unreliable, leading to inappropriate transport decisions (26, 83). For these patients, paramedics are advised to administer smaller fluid volumes with careful monitoring to avoid fluid overload (3).

The reliability of the Glasgow Coma Scale (GCS) is also debated for older patients with head injuries. Studies have shown that older patients with intracranial bleeding may present with normal or near-normal GCS scores, and a score of 14 or less is suggested as an appropriate threshold for transport to a major trauma centre (MTC) (36, 51). However, diminished hearing and environmental noise may affect paramedics’ ability to accurately determine GCS scores at the scene (36). Lastly, older patients are at increased risk of hypothermia and infections due to immune senescence and premorbid malnutrition, requiring additional consideration when managing the exposure component of the primary survey (48).

### 5.3 When triaging older trauma patients and selecting a definitive care facility

Effective triage is essential in determining appropriate hospital destinations for trauma patients and in ensuring optimal use of trauma system resources (27, 76). For older adults, this process is particularly complex due to atypical presentations, multiple comorbidities, and age-related physiological decline (77). Traditional trauma triage tools such as the US Field Triage Decision Scheme and the Ohio Trauma Triage Protocol that were primarily developed based on data from younger populations, which limits their accuracy in identifying serious injuries in older adults (10, 28, 29, 76). The US Field Triage Decision Scheme follows a four-step process incorporating physiological, anatomical, mechanism of injury, and special considerations (30). While this tool is widely used, it has shown reduced sensitivity in older adults, increasing the risk of undertriage (27). The Ohio Trauma Triage Protocol includes geriatric-specific adaptations such as a lower systolic blood pressure (SBP) threshold and modified GCS criteria (31), resulting in significantly improved sensitivity for identifying older trauma patients requiring higher-level care (10). However, these modifications may lead to increased overtriage and strain on trauma centres (10). Table 1 shows these protocols, previously summarised by Harthi et al. (76), and based on recent evidence (10, 27).

**TABLE 1:** Traditional prehospital trauma triage tools for older adults: features, supporting evidence, and limitations.

Triage tool	Key features	Evidence	Limitations
US Field Triage Decision Scheme	Four-step triage process: 1. Physiological: GCS < 13, SBP < 90 mmHg, abnormal respiratory rate (RR) 2. Anatomical: penetrating injuries, long-bone fractures, skull/pelvic fractures 3. Mechanism of injury: falls > 20 ft, vehicle ejection, pedestrian struck 4. Special considerations: age ≥55, anticoagulant use, pregnancy, clinical judgement -Designed to optimise use of trauma system resources	-Evaluated across 94 EMS agencies and 122 hospitals -Sensitivity: 85.8% (overall), 79.9% in older adults -Specificity: 68.7% -Final triage including hospital input improved sensitivity to 82.6%	-Lower sensitivity in older adults, raising undertriage risk -Steps 1 and 2: high specificity but lower sensitivity -Steps 3 and 4: improve sensitivity but increase overtriage -Lacks frailty or comorbidity assessment -No standardised geriatric-specific modifications implemented
Ohio Trauma Triage Protocol	-Three main components: 1. Physiological: GCS ≤ 13, SBP < 90 mmHg, RR < 10 or >29, loss of consciousness > 5 min 2. Anatomical: penetrating injuries, flail chest, spinal cord injury, pelvic fractures, amputations, burns >10% TBSA 3. Additional considerations: mechanism of injury, age, comorbidities -Geriatric-specific adjustments (2009): •Raised SBP threshold to <100 mmHg •GCS ≤ 14 for TBI •Single long-bone fracture •Standing falls with TBI •Multiple body region injuries	-Analysed data from 101,577 patients (33% geriatric) -Geriatric criteria sensitivity: 93% (vs. 61% adult criteria) -Specificity: 49% (vs. 61%) -Improved detection of ICU admission, mortality, and surgery needs in older adults	-Reduced specificity leads to increased overtriage -May strain trauma centres due to more non-critical patients being referred -Developed for Ohio—limited generalisability to other regions -Does not include frailty screening despite age-adjusted criteria -Requires further validation in diverse EMS systems

Despite these efforts, neither protocol includes formal frailty assessment, which has emerged as a crucial factor in predicting outcomes among older trauma patients (52, 76). Tools such as the Clinical Frailty Scale (CFS), Programme on Research for Integrating Services for the Maintenance of Autonomy (PRISMA-7), and Identification of Seniors at Risk (ISAR) have shown promising predictive value for mortality, hospitalisation, and functional decline (76, 80). PRISMA-7 is a short, self-reported questionnaire that demonstrates high sensitivity and specificity in emergency settings (76, 80). The CFS offers a clinician-rated frailty score based on physical function and comorbidities, while ISAR is designed to identify older patients at risk of adverse outcomes during ED or hospital admission (76, 80). These tools, although validated in hospital environments, are not yet widely implemented in prehospital triage protocols (52, 76). Barriers include time constraints, lack of training, and the absence of EMS-specific adaptations (1, 75). Nevertheless, integrating frailty assessments into prehospital triage could significantly improve risk stratification, reduce undertriage, and enhance decision-making for older trauma patients (76). Table 2 presents these frailty assessment tools, which were previously summarised by Harthi et al. (76), and are based on recent evidence (32, 53, 73, 80, 82).

**TABLE 2:** Frailty assessment tools in emergency and trauma care: key features, diagnostic accuracy, and prehospital applicability.

Tool	Key features	Evidence of predictive accuracy	Strengths	Limitations/barriers to prehospital use
PRISMA-7 (Programme on Research for Integrating Services for the Maintenance of Autonomy)	-7-item questionnaire -Self-reported -Screens for disability and comorbidity	-AUC: 0.88 -Sensitivity: 84%, Specificity: 78% -Best at distinguishing pre-frail vs. frail (AUC: 0.71)	-High diagnostic accuracy -Short administration time -Strong inter-rater reliability (*r* = 0.75) -Effective in ED triage settings	-Not yet validated for EMS use -Dependent on patient response -No digital tool for EMS application -Requires further feasibility testing in prehospital settings
CFS (Clinical Frailty Scale)	-9-point clinical judgement scale -Based on functional status and comorbidities -Requires trained rater	-AUC: 0.83 -Validated in trauma patients -Strong predictor of 30-day mortality and adverse outcomes	-Greater specificity than ISAR or PRISMA-7 -Predicts mortality, delirium, and discharge needs -Widely used in EDs across multiple countries	-Moderate inter-rater reliability (*r* = 0.78) -Requires clinical training -Longer assessment time than PRISMA-7 -Not routinely used in prehospital triage
ISAR (Identification of Seniors at Risk)	-6-item screening tool -Self-reported -Focuses on prior hospital use, ADLs, memory	-AUC: 0.78 -Sensitivity: 95%, specificity: 35% -Highest sensitivity, but lowest specificity	-Quick to administer -High sensitivity -Suitable for initial risk identification	-Poor specificity → high false-positive rate -Weakest diagnostic accuracy overall -Reliability lower (*r* = 0.62) -May overburden trauma centres if used without refinement

### 5.4 During secondary survey

During the secondary survey, paramedics gather the patient’s history, perform a physical examination, and monitor vital signs (49). Ageing can limit communication, as diminished hearing may hinder paramedics from obtaining crucial information regarding chronic conditions, prescribed medications, and the mechanism of injury (36, 65). Older adults may also have reduced pain perception, making it challenging for paramedics to identify injuries during the physical examination (3). Furthermore, traditional indicators like pulse rate and blood pressure may be misleading due to medication effects, such as antihypertensives or pacemakers, which can mask signs of injury severity (83). It is, therefore, critical for paramedics to gather detailed information on polypharmacy during the secondary survey to ensure effective care (3).

### 5.5 During transport to hospital

Transporting older trauma patients presents its own set of challenges. Ideally, patients with major trauma should be taken directly to an MTC, while those with less severe injuries should be transported to a trauma unit (54). Paramedics must consider that older adults are vulnerable to high-risk injuries even with low-impact mechanisms, such as ground-level falls (36, 51). Fragility fractures, hidden hypoperfusion, and life-threatening injuries like intracerebral bleeding may remain unrecognised during prehospital care (3, 36, 51).

Under-triage of older trauma patients, resulting in transport to non-MTCs, is often attributed to a lack of paramedic training or awareness of age-specific issues in trauma care (24, 33, 36). This failure in appropriate triage increases the risk of deterioration en route and can lead to higher mortality rates (36). Hildebrand et al. two studies recommended proactive measures for paramedics, including early monitoring, thorough physical assessment, aggressive resuscitation, timely management of injuries, and rapid haemostasis (4, 48). These strategies, adapted for ageing-related changes, can help reduce mortality risk in older trauma patients.

## 6 The role of paramedics in providing geriatric trauma care and understanding ageing-related impacts

Assessing frailty accurately is a significant challenge within hospital settings, where the time and resources required may not be available (66). Detailed information regarding the patient’s living environment, social support, and medical history may be lacking if relatives or other carers are not present (66). However, ambulance personnel can bridge this gap. During emergency calls, paramedics are present in the patient’s home, allowing them to observe important environmental clues and gain insights into the patient’s daily life, family dynamics, and social situation (67, 68).

This extended interaction provides paramedics with opportunities to collect essential information that might be missed during hospital admission (66). Information gathered at the scene can significantly influence decisions made in the emergency department regarding treatment goals, the need for diagnostic tests, social care, and discharge planning (66). Therefore, the role of paramedics extends beyond stabilisation and transport; it also encompasses initiating a comprehensive care process that addresses both medical and social determinants of health (69).

The insight provided by paramedics helps to ensure that older adults receive more tailored care, potentially improving overall outcomes. For instance, paramedics can detect frailty, understand the patient’s baseline functional status, and identify any signs of elder abuse, all of which can impact the course of treatment (3, 24). In light of these unique challenges, enhanced training for paramedics on geriatric assessment and trauma care is essential to improve outcomes for older adults (33, 34).

## 7 Enhancing paramedic awareness of the impacts of ageing changes to improve geriatric trauma care

In the context of geriatric trauma care, enhancing paramedic awareness of ageing-related changes is crucial for improving patient outcomes (55). Current evidence suggests that insufficient knowledge and awareness among ambulance clinicians regarding ageing impacts remain significant barriers to optimal care. For instance, paramedics often lack understanding of how age-related physiological changes, such as polypharmacy effects (e.g., anticoagulants and antiplatelets), influence trauma outcomes, especially in older patients with TBI (35, 37). This lack of awareness increases the risk of adverse outcomes, such as excessive bleeding, and contributes to undertriage in prehospital care (33, 34). Further research is needed to explore factors contributing to this knowledge gap, including the decision-making processes and behaviours of paramedics when managing older patients (55).

Inadequate training in geriatric-specific care further exacerbates these challenges. Paramedics in Saudi Arabia frequently reported insufficient education on ageing-related changes during university studies and limited access to relevant training courses (75). This knowledge gap results in difficulties in applying essential skills, such as communication, predicting adverse outcomes, and performing technical tasks like IV cannulations (75). Similarly, studies from Canada and the United States indicate that prehospital providers acquire limited geriatric-specific knowledge, leaving them less prepared to address the unique needs of older patients compared to their in-patient counterparts (38, 66). Additionally, cultural factors may hinder effective communication and application of knowledge, as observed among paramedics in the United Kingdom and Saudi Arabia (56, 75).

Training interventions are essential to address these gaps (1, 55, 75). Actions such as incorporating geriatric-specific education into prehospital curricula, providing ongoing training on ageing-related challenges, and familiarising paramedics with the long-term impacts of injuries in older patients are critical (38, 55, 70, 75). Furthermore, targeted training on topics like polypharmacy risks, frailty assessment, field-triage criteria, and holistic care for older patients can significantly enhance paramedics’ readiness to manage geriatric trauma (29, 33, 37, 39–41, 78, 85). Training should also address behavioural and cultural aspects of care to improve communication and foster a safety-oriented working culture (75).

The importance of improving paramedic knowledge and attitudes towards geriatric care cannot be overstated (55). Studies have shown that paramedics often perceive falls and low-mechanism injuries among older adults as less severe, leading to underestimation of their clinical significance (74, 75). By enhancing paramedic education and addressing misconceptions, emergency medical services can ensure better triage, reduce undertriage, and ultimately improve outcomes for older patients (29, 41, 78). This shift requires a sustained commitment to integrating geriatric principles into prehospital care, accompanied by robust research to identify and address the underlying causes of knowledge deficits (55).

## 8 Conclusion

The increasing proportion of older adults in the global population presents significant challenges for prehospital trauma care, particularly as ageing-related physiological changes profoundly influence clinical outcomes. This narrative review has explored how such changes, affecting the nervous, cardiovascular, respiratory, musculoskeletal, and renal systems that complicate trauma assessment and management in the prehospital setting. These challenges are further exacerbated by frailty, polypharmacy, and comorbidities, which demand more nuanced and individualised approaches to care, especially during initial assessment, triage, and transport.

Despite the increasing burden of trauma among older adults, gaps persist in paramedics’ knowledge and preparedness to manage age-related complexities effectively. Traditional trauma triage tools frequently lack sensitivity and fail to account for frailty, leading to undertriage and inappropriate care decisions. Although some systems, such as the Ohio Trauma Triage Protocol, incorporate geriatric-specific criteria, they still do not address the broader functional and physiological diversity of older patients. This underscores the importance of integrating validated frailty assessment tools such as the CFS, PRISMA-7, and ISAR into prehospital triage protocols.

Improving outcomes for older trauma patients requires targeted interventions, including geriatric-specific education, ongoing professional development, and tailored training on frailty, polypharmacy, and decision-making under uncertainty. Strengthening paramedics’ ability to recognise and respond to ageing-related changes is essential for enhancing both triage accuracy and clinical care.

Future research should focus on refining geriatric triage models, validating frailty tools in EMS contexts, and exploring how paramedics’ clinical judgements are influenced by patient age, presentation, and situational factors. Investigating EMS providers’ decision-making behaviours and cultural attitudes towards older adults will help identify modifiable training gaps and system-level barriers. By addressing these issues through education, clinical guidance, and research-driven protocols, prehospital care systems can become more responsive, equitable, and effective for the ageing trauma population.
