# Correction: The impacts of ageing-related changes on prehospital trauma care for older adults: challenges and future directions

**DOI:** 10.3389/fmed.2025.1657796

**Published:** 2025-07-17

**Authors:** Naif Harthi, Steve Goodacre, Fiona C. Sampson

**Affiliations:** 1 Emergency Medical Services Programme, Department of Nursing, College of Nursing and Health Sciences, Jazan University, Jazan, Saudi Arabia; 2 Health Research Center, Jazan University, Jazan, Saudi Arabia; 3 Sheffield Centre for Health and Related Research (SCHARR), University of Sheffield, Sheffield, United Kingdom

**Keywords:** ageing changes, ageing impacts, trauma, prehospital care, older people

In the published article, there were errors in the author affiliations.

Missing Affiliation for Author Naif Harthi

Dr. Naif Harthi was only listed with one affiliation. The following affiliation was erroneously omitted:

Health Research Center, Jazan University, Jazan, Saudi Arabia

This affiliation has now been added for Dr. Naif Harthi. He is correctly affiliated with:

1. Emergency Medical Services Programme, Department of Nursing, College of Nursing and Health Sciences, Jazan University, Jazan, Saudi Arabia

2. Health Research Center, Jazan University, Jazan, Saudi Arabia

Incorrect Affiliations for Authors Steve Goodacre and Fiona C. Sampson

Professor Steve Goodacre and Dr. Fiona C. Sampson were incorrectly assigned to Affiliation 2. Their correct affiliation is:

Sheffield Centre for Health and Related Research (SCHARR), University of Sheffield, Sheffield, United Kingdom

They should both be affiliated with this institution only.

Reviewer “**Łukasz Dudziński**” has changed affiliation to the Medical University of Warsaw. In the published article their previous affiliation was incorrectly listed as State School John Pope Biala Podlaska.

The original version of this article has now been updated.

